# Achalasia is associated with a higher incidence of depression in outpatients in Germany

**DOI:** 10.1371/journal.pone.0250503

**Published:** 2021-04-30

**Authors:** Sven H. Loosen, Jennis Kandler, Tom Luedde, Karel Kostev, Christoph Roderburg

**Affiliations:** 1 Clinic for Gastroenterology, Hepatology and Infectious Diseases, University Hospital Düsseldorf, Medical Faculty of Heinrich Heine University Düsseldorf, Düsseldorf, Germany; 2 Epidemiology, IQVIA, Frankfurt, Germany; Chiba Daigaku, JAPAN

## Abstract

**Background and aim:**

Achalasia represents a chronic motility disorder of the esophagus featuring an impaired lower esophageal sphincter relaxation and loss of esophageal peristalsis. By causing dysphagia, regurgitation, aspiration and chest pain, achalasia might tremendously affect life quality of patients. However, the impact of achalasia on the development of mood disorders including depression has largely remained unclear. The aim of this study was to evaluate the incidence of depression in achalasia patients.

**Methods:**

We analyzed a large primary care cohort database in Germany capturing data from 7.49 million patients.

**Results:**

A total of n = 1,057 patients with achalasia diagnosed between January 2005 and December 2018 were matched to a cohort of n = 3,171 patients without achalasia controlling for age, sex, physician, index year, and the Charlson comorbidity index. Interestingly, while the frequency of depression prior to the diagnosis of achalasia was comparable in both groups, new diagnoses of depression were significantly higher within one year after the diagnosis of achalasia compared to the control group, suggesting a direct and previously unrecognized association between achalasia and depression.

**Conclusion:**

Our data suggest that the clinical management of patients with achalasia should include a careful and structured work-up for mood disorders in order to improve long-term quality of life in these patients.

## Introduction

The first description of achalasia goes back to Sir Thomas Willis in 1674. Today, achalasia is defined as a chronic disorder of esophageal motility characterized by an impaired lower esophageal sphincter (LES) relaxation and the loss of esophageal peristalsis [1]. Achalasia is an exceptionally rare disease. Different studies estimate the incidence at 1.6 per 100,000 population and the prevalence at 10.8 per 100,000 population. Males and females are equally affected, most patients are diagnosed between 30 to 60 years of age [2, 3]. Only about 3% of cases occur in children [2].

Achalasia is characterized by a lack of relaxation of the lower esophageal sphincter. There is often also a disturbance in the peristaltic contractions of the esophageal muscles. In approximately 50% of cases, there is a fixed hypertensive contraction of the lower esophageal sphincter. All of these pathologies lead to the pathognomonic functional obstruction at the gastroesophageal junction. The specific pathophysiology of achalasia is only poorly understood. Different studies suggested that achalasia might occur due to a degeneration of the myenteric plexus and vagus nerve fibers of the lower esophageal sphincter [4, 5]. This might occur as a result of autoimmune diseases, viral infections, neurodegenerative disorders, eosinophilic gastroenteritis, or esophageal infiltration by gastric carcinoma [6].

The goal of achalasia treatment is to relieve the symptoms caused by the disorder. Both, surgical (e.g. myotomy and peroral endoscopic myotomy (POEM)) as well as non-surgical procedures are available [7–9]. However, in some patients, optimal symptom control cannot be achieved, leaving these with long-term distress symptoms. We therefore hypothesized that this distress could be associated with an increased incidence of depression and anxiety disorders in achalasia patients as recently demonstrated for other esophagus related diseases [10–12]. Due to the rarity of the disease, such a question can only be answered by analyzing large patient registries. We therefore used a primary care provider database covering 7.5 million health data from Germany [13, 14], to analyze an association of depression in patients with achalasia compared to matched controls as recently described (e.g. [15]).

## Methods

### Database

This study used data from the Disease Analyzer database (IQVIA). Full details of the database have been published before [14]. Briefly, the Disease Analyzer database is composed of sociodemographic, diagnosis, and prescription data obtained in general and specialized practices in Germany. Diagnosis data are based on the German adaptation of the International Classification of Diseases, 10^th^ revision (ICD-10), while prescription data are coded using the European Pharmaceutical Marketing Research Association (EphMRA) Anatomical Therapeutic Chemical (ATC) classification system. The quality of the data is assessed regularly by IQVIA based on a number of criteria (e.g., completeness of documentation and linkage between diagnoses and prescriptions). The database includes only anonymized data in compliance with the regulations of the applicable data protection laws. It has previously been found that the panel of practices included in the Disease Analyzer database is representative of general and specialized practices in Germany [14]. The "Disease Analyzer" database contains anonymized electronic patient records. Patient data was analyzed in aggregated form without individual health data being available. An individual consent form was not obtained.

### Study population and variables

This mixed retrospective case-control and cohort study included adult outpatients (≥18 years) with an initial diagnosis of achalasia (ICD-10: K22.0) in 1,262 general practices in Germany between January 2005 and December 2018 (index date). Patients with achalasia were matched to patients without achalasia 1:3 by age, sex, physician, index year, obesity diagnosis, and Charlson comorbidity index (CCI). The CCI is a weighted index that accounts for the number and severity of comorbidities in administrative database studies and includes a wide range of comorbidities (e.g. macrovascular diseases, pulmonary diseases, gastrointestinal diseases, liver and renal diseases, diabetes, tumors, and acquired immune deficiency syndrome [16]).

### Study outcomes and covariates

The main outcome of the study was the cumulative incidence of depression (ICD-10: F32, F33) as a function of achalasia. Depression incidence was estimated in three time periods: within one year prior to the index date, within more than one year prior to the index date, and within one year after the index date. Additionally, the proportion of depression patients with an antidepressant prescription was estimated.

### Statistical analyses

Differences in the sample characteristics between those with and those without achalasia were tested using chi-squared tests for categorical variables and Wilcoxon tests for continuous variables. The cumulative incidence of depression was estimated in three time periods and compared between patients with and without achalasia using chi-squared tests. Univariate logistic regression analyses were further conducted to investigate the association between achalasia and depression in three time periods. Results from the logistic regression analyses are presented as odds ratios (ORs) with 95% confidence intervals (95% CIs). Both descriptive analyses and logistic regression analyses were performed for total cohorts and separately for men and women, as well as five age groups. In regression analyses, p-values were corrected using the Bonferroni adjustment method and were considered statistically significant at < = 0.006 calculated as 0.05/8 (8 comparisons). Analyses were carried out using SAS version 9.4 (SAS Institute, Cary, USA).

## Results

### Cohort characteristics

**Fig 1:**
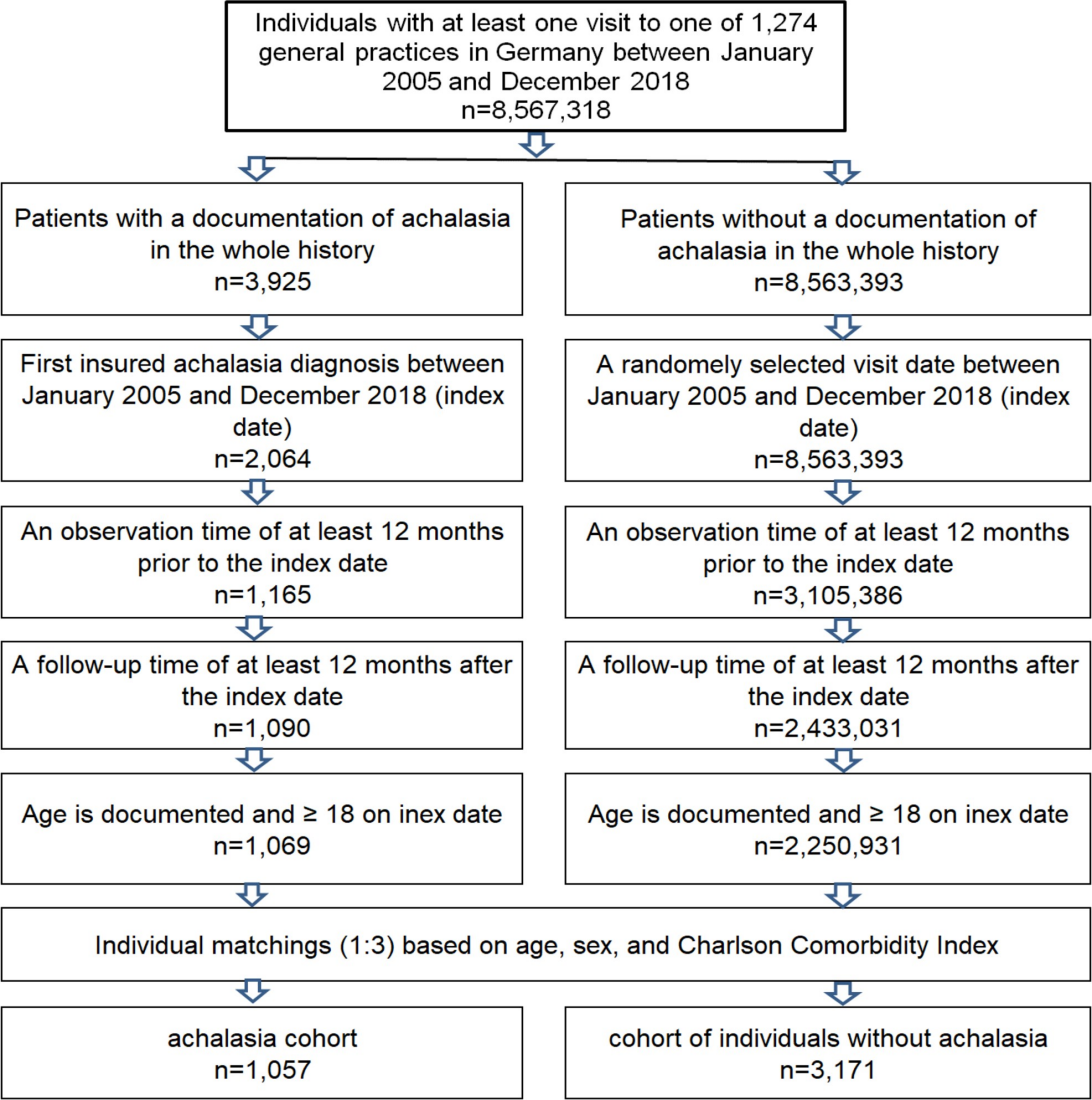
Selection of study patients.

We included a total of n = 1057 patients as well as n = 3171 matched patients without achalasia (Fig 1). Detailed patient characteristics are given in Table 1. 46.9% of patients were male, 53.1% of patients were female. The mean age was 61.9 years (SD 16.1), the mean CCI was 2.6 (SD 2.3). The patients were distributed in a balanced way over the different age groups, with a maximum in the age group of patients between 51–60 years and 71–80 years (23.9% of patients each, Table 1).

**Table 1:** Baseline characteristics of study patients after 1:3 matching.

Variable	Patients with achalasia (N = 1,057)	Patients without achalasia (N = 3,171)	P-value
*Sex*			
Men	46.9	46.9	1.000
Women	53.1	53.1	
*Age*			
Mean age in years (standard deviation)	61.9 (16.1)	61.9 (16.1)	1.000
Age 18–50 years	21.4	21.4	1.000
Age 51–60 years	23.9	23.9	
Age 61–70 years	18.2	18.2	
Age 71–80 years	23.9	23.9	
Age >80 years	12.5	12.5	
*Charlson Comorbidity Index (mean*, *SD)*	2.6 (2.3)	2.6 (2.3)	1.000

Data are absolute numbers and percentages unless otherwise specified.

### Incidence of depression and antidepressant therapy before diagnosis of achalasia

**Fig 2:**
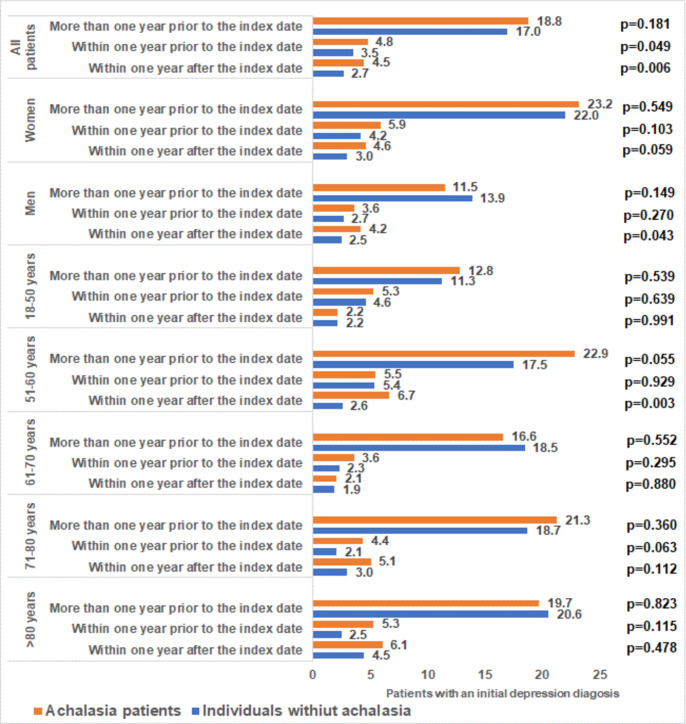
Cumulative incidence of documented depression among individuals with and without achalasia diagnoses followed in general practices.

We first compared the incidence of depression between achalasia patients and matched controls more than one year prior to the index data as well as within the last year prior to the index date. There were small differences between the two groups (18.8% vs. 17.0%, p = 0.181; and 4.8% vs. 3.5%, p = 0.049) (Fig 2). In the time period more than one year prior to the index date, 13.0% vs. 8.0% (p<0.001) received antidepressant prescription; within one year prior to the index date, 1.4% vs. 1.2% (p = 0.626) received antidepressants. In regression analyses, no significant association was observed between achalasia and depression diagnosis in both time periods prior to the index date (OR: 1.13, 95% CI: 0.95–1.35 for more than one year prior to the index date, and OR: 1.40, 95% CI: 1.00–1.97 for within one year prior to the index date). No significant associations were seen in sex- and age-subgroups (Table 2). However, achalasia was significantly associated with antidepressant prescription in the time period more than one year prior to index date (OR: 1.70, 95% CI: 1.37–2.12).

**Table 2:** Association between achalasia and depression in patients followed in general practices in the Germany.

	More than one year prior to the index date		Within one year prior to the index date		Within one year after the index date	
Population	OR (95% CI)*	P-value**	OR (95% CI)*	P-value**	OR (95% CI)*	P-value**
Overall	1.13 (0.95–1.35)	0.181	1.40 (1.00–1.97)	0.050	**1.66 (1.15–2.38)**	**0.006**
Women	1.07 (0.85–1.35)	0.549	1.42 (0.93–2.17)	0.105	1.59 (0.98–2.58)	0.061
Men	1.25 (0.92–1.68)	0.149	1.37 (0.78–2.42)	0.271	1.75 (1.01–3.01)	0.045
Age 18–50 years	1.16 (0.73–1.83)	0.532	1.18 (0.59–2.33)	0.640	1.01 (0.36–2.80)	0.991
Age 51–60 years	1.41 (0.99–1.99)	0.056	1.02 (0.55–1.92)	0.929	**2.69 (1.38–5.18)**	**0.004**
Age 61–70 years	0.88 (0.57–1.35)	0.552	1.64 (0.64–4.17)	0.300	1.09 (0.34–3.47)	0.880
Age 71–80 years	1.18 (0.83–1.68)	0.360	2.12 (0.97–4.64)	0.059	1.75 (0.87–3.50)	0.117
Age >80 years	0.95 (0.58–1.55)	0.823	2.17 (0.81–5.83)	0.123	1.36 (0.58–3.21)	0.480

*OR = Odds Ratio, 95% CI = 95% confidence intervals.

**p< = 0.006 is considered statistically significant (adjusted for Bonferroni).

### Incidence of depression and antidepressant therapy after diagnosis of achalasia

Within 1 years of the index date, 4.5% of patients with achalasia and 2.7% of individuals without achalasia were diagnosed with depression (p = 0.006, Fig 2). In addition, 1.1% vs. 0.9% received an antidepressant prescription (p = 0.456). In regression analyses, achalasia was significantly associated with the incidence of depression (OR: 1.66, 95% CI: 1.15–2.38). In age- and sex-stratified analyses, however, the association was only significant in the age group 51–60 years (OR: 2.69, 95% CI: 1.38–5.18). There was no significant association between achalasia and antidepressant prescription.

## Discussion

In this study, we demonstrate that patients with achalasia, after the diagnosis of achalasia, develop depression at significantly higher rates than patients without achalasia. Of note, this effect was observed both in males and females. In line to our results, in a recent analysis, patients with gastroesophageal reflux disease, which might display similar symptoms than achalasia were demonstrated to exhibit psychological symptoms at higher rates than controls. In this analysis, long symptom duration turned out as a risk factor for development of depression, highlighting that esophagus related diseases might be associated to depressive disease states as recently demonstrated (e.g. [10–12]).

Achalasia represent a very rare disease with a prevalence of about 10 cases per 100,000 inhabitants [2]. To examine an association between achalasia and depression, we used a primary care provider database covering over 7.5 million health data records with a coded prevalence of 0.046%, which is somewhat higher than the previously reported values. Out of the 3925 patients with achalasia in the database, n = 1057 fulfilled the criteria for further analysis (see Fig 1) and were matched according to their age, sex and CCI with a 1:3 ratio to n = 3171 controls.

Achalasia most probably results from a loss of the myenteric plexus and vagus nerve fibers of the lower esophageal sphincter [5]. It has been hypothesized that achalasia is not a mono-causal disease involving only one pathway but rather results from a combination of infectious, autoimmune, and genetic components [17]. Of note, all of these have been linked to psychiatric disorders such as anxiety and depression. As an example, histopathological studies have revealed the degeneration of neurons that are part of the cholinergic system [6], which is also involved in the regulation of emotion and motivation [18]. In line, Duarte-Silva et al. have recently described shared neuroimmune and oxidative pathways underpinning Chagas disease and major depressive disorder, providing a direct link between the pathophysiology of achalasia and depressive disorders [19]. Additionally, the loss of life quality due to achalasia might be tremendous, with dysphagia to solids and liquids, regurgitation, weight loss and chest pain representing the most important symptoms [20]. Despite recent data, linking achalasia and depression on a pathophysiological level, it is important to note that our data do not allow to differentiate between reactive depression and idiopathic depression caused by pathways common to achalasia and depressive disorders. Thus, further studies are warranted to further elucidate the association between achalasia and mood disorder, described in our study.

Our study has several limitations: Despite the German Disease Analyzer database used here has been validated in several medical studies [14], the conducted analysis relies on ICD-10 codes for establishing diagnoses, which may cause misclassification bias. Moreover, the database did not include (longitudinal) data on patients’ symptoms and potential alleviation of these symptoms due to treatment. Thus, we cannot exclude that the development of depression is restricted to patients failing to respond to treatment, and that- in turn- a successful treatment of achalasia might be prevent depression in these patients. In this context it is important to note that success rates of the different treatments for achalasia vary between 20 and 80%, leaving many patients symptomatic despite optimal treatment. Furthermore, we had no data on factors known to have a significant impact on mental health (e.g., loneliness, social support, alcohol use), and this may have biased our findings. Moreover, this was a retrospective study, and it was thus not possible to determine causality in the association between achalasia and depression. Finally, it is important to note that we cannot exclude a selection bias in our study for those with achalasia diagnosis. It seems possible that patients who have an established diagnosis of achalasia may have higher levels of care-seeking behavior and are therefore more likely to be screened for depression.

In summary, our study is the first using a primary care provider database to demonstrate that achalasia is associated with an increased incidence of depression irrespective of other comorbidities or patients’ characteristics. Our data suggest that the clinical management of patients with achalasia should include a careful and structured work-up for mood disorders in order to improve long-term quality of life for these patients.
